# Water recycling by Amazonian vegetation: coupled versus uncoupled vegetation–climate interactions

**DOI:** 10.1098/rstb.2007.0035

**Published:** 2008-02-11

**Authors:** S.A Cowling, Y Shin, E Pinto, C.D Jones

**Affiliations:** 1 Department of Geography, University of Toronto100 St George Street, Toronto, Ontario, Canada M5S 3G3; 2 Faculty of Forestry, University of Toronto33 Wilcocks Street, Toronto, Ontario, Canada M5S 3B3; 3 Met Office, Hadley Centre for Climate Prediction and ResearchFitzroy Road, Exeter, Devon EX1 3PB, UK

**Keywords:** Amazon Basin, water recycling, evapotranspiration

## Abstract

To demonstrate the relationship between Amazonian vegetation and surface water dynamics, specifically, the recycling of water via evapotranspiration (ET), we compare two general circulation model experiments; one that couples the IS92a scenario of future CO_2_ emissions to a land-surface scheme with dynamic vegetation (coupled) and the other to fixed vegetation (uncoupled). Because the only difference between simulations involves vegetation coupling, any alterations to surface energy and water balance must be due to vegetation feedbacks. The proportion of water recycled back to the atmosphere is relatively conserved through time for both experiments. Absolute value of recycled water is lower in our coupled relative to our uncoupled simulation as a result of increasing atmospheric CO_2_ that in turn promotes lowering of stomatal conductance and increase in water-use efficiency. Bowen ratio increases with decreasing per cent broadleaf cover, with the greatest rate of change occurring at high vegetation cover (above 70% broadleaf cover). Over the duration of the climate change simulation, precipitation is reduced by an extra 30% in the coupled relative to the uncoupled simulations. Lifting condensation level (proxy for base height of cumulus cloud formation) is 520 m higher in our coupled relative to uncoupled simulations.

## 1. Introduction

Over the past decade, the role of vegetation in modifying atmospheric processes has become increasingly the focus of Earth science investigations; incorporating methods involving modelling of land-use change and vegetation–climate feedbacks, remote sensing and observations (measurements) of atmospheric and land-surface variables (Crucifix *et al*. 2005; Gedney *et al*. 2006; Henderson-Sellers 2006; Salmun & Molod 2006; Pitman & Hesse 2007). Without debate, the terrestrial biosphere is an integral component of the Earth system; only now we are realizing the extent to which terrestrial vegetation can alter the exchange of carbon, water and energy within the active boundary layer. The structure of vegetation, for example, can alter the reflectivity of the Earth's surface (albedo), thereby modifying surface radiation balance (Da Silva & Avissar 2006). The physiology of plant canopies (i.e. stomata) influences canopy water exchange (Roberts 2007). Stomata are also critical for carbon exchange between the biosphere and the atmosphere and are influenced by both external forces such as microclimate and internal factors such as plant stress hormones.

Owing to the increasing awareness of vegetation feedbacks on climate (in particular, the consequence of deforestation for regional climate) (Pielke *et al*. 1998), Amazonia has become a popular region for study. The LBA project (Avissar *et al*. 2002) has provided a girth of observational (eddy-flux) data on canopy–atmosphere gas exchange. One area of popular interest, owing to its continuing uncertainty, is the degree to which canopies (i.e. cumulative plant stomata) can alter surface water balance that in turn may influence larger-scale processes, such as lifting condensation level (LCL; cloud base height) and rate of precipitation.

It was the objective of our research to evaluate pre-existing model simulations of coupled and uncoupled vegetation feedbacks on climate for insights into the potential role that plant physiology has in modifying surface water balance. Physiological alterations to surface water may arise from two plant-based responses: (i) increases (decreases) in leaf surface area that concurrently increase (decrease) rates of transpiration and (ii) decreases (increases) in stomatal conductance that modify pore openings and therefore control the amount of transpired water. We evaluate simulated results including per cent broadleaf cover, evapotranspiration (ET), precipitation and surface energy balance (Bowen ratio) from two simulations with HadCM3LC, a climate–carbon cycle configuration of the Met Office Unified Model. The first simulation was conducted using the IS92a CO_2_ emission scenario, incorporating a fixed biosphere (uncoupled), while the other simulation was conducted with the same climate forcing variables but incorporating a dynamic biosphere module (coupled).

We can insightfully compare these simulations because the only difference between simulations is whether or not the biosphere feedbacks onto climate-modifying variables. The same experiment could be run with one (uncoupled) GCM model simulated with different fixed vegetation fractions (i.e. 100, 80, 60% broadleaf cover, etc.). We chose our methodological approach for two reasons: (i) simulation results already existed from previous simulation experiments (Cox *et al*. 2004) and (ii) we are able to evaluate transient vegetation–climate interactions through time, thus avoiding ‘snap shot’ assessments.

Understanding the consequences of changing vegetation cover for surface water budget is important for better understanding the role that vegetation feedbacks have on larger-scale processes such as cumulus cloud formation and rates of precipitation (Eltahir & Bras 1996). Therefore, we also compare the effects of coupled and uncoupled vegetation feedbacks on LCL and the parameters that underlie cumulus cloud formation (i.e. specific humidity, surface temperature and surface pressure).

## 2. Material and methods

### (a) Model description

HadCM3LC is the Hadley Centre's fully coupled atmosphere–ocean GCM. It consists of HadCM3 (Gordon *et al*. 2000) coupled to an ocean carbon cycle model HadOCC (Palmer & Totterdell 2001) and a dynamic global vegetation model, TRIFFID (Cox 2001). HadCM3LC has a resolution of 2.5° latitude by 3.75° longitude, 19 vertical atmospheric levels (employing a hybrid vertical coordinate system) and a computational time step of 30 min. The model was developed over a decade ago; hence has undergone rigorous testing of its parametrizations and mechanistic algorithms, as well as been employed in numerous Earth system applications (Betts *et al*. 2004; Jones *et al*. 2005; Toniazzo 2006; Grist *et al*. 2007; Küttel *et al*. 2007; Lachlan-Cope *et al*. 2007). To avoid unnecessary repetition, we direct the reader to refer to one of these aforementioned studies to obtain more detailed information about the Hadley GCM.

### (b) Model simulation protocols

Climate boundary conditions were similar for both scenarios and based on IPCC's IS92a trend in CO_2_ emissions to the atmosphere (Cox *et al*. 2000, 2004). Vegetation in the uncoupled scenario is fixed at approximately 84% broadleaf cover, whereas the coupled simulation shows a progressive decrease in per cent broadleaf cover from 84% near the start of the simulation (*ca* AD 2000) to just above 50% near the end of the simulation (*ca* AD 2090; figure 1*a*). This decrease demonstrates the dieback of Amazonian rainforest in response to a warmer and drier Basin. The GCM simulation is actually begun at the start of the Industrial Revolution (AD 1875) so that by the simulation year AD 2000, the influence of vegetation on regional climate can already be detected. We chose to begin our analysis at the year AD 2000 because it is at this point that we can evaluate the influence of vegetation on climate interactions under modern-day [CO_2_] and climate forcing.

We extracted model output variables as they relate to water exchange at the canopy (i.e. per cent broadleaf cover, ET, and latent and sensible heat) as well as variables indicating larger-scale water balance processes, such as precipitation and LCL. The latter variable (LCL) was calculated using GCM-derived variables, such as specific humidity, surface pressure and temperature.

### (c) Comparison of modelled results against field observations of surface water balance

We have produced a summary of observations from some recent field research in the tropics, including Amazonia. These data include eddy-flux calculations of canopy–atmosphere exchange. Such comparison of modelled values (at AD 2000) against observations will help gauge the relative ‘reliability’ of HadCM3LC in predicting canopy–atmosphere water exchange parameters, in specific the degree to which vegetation recycles water via transpiration. We define water-recycling efficiency (WRE) in terms of ET and precipitation (P) such that WRE = ET/P.

### (d) Comparison of modelled results against observational data of LCL in Amazonia

Because there is much concern in the literature about the inability of GCMs to accurately capture surface hydrological processes (Still *et al*. 1999; Henderson-Sellers 2006), we performed a basic comparison of model-predicted LCL for modern-day climate with observations of LCL recorded over the Amazonian Basin (Pinto *et al*. in press). Observed LCL data were retrieved from a compilation study by Hahn & Warren (2002) that contained three-month-averaged cumulus cloud base heights measured between 1971 and 1996 (at a resolution of 5° latitude by 5° longitude). Our GCM-derived LCL values are annual means therefore a *direct* comparison between observed and calculated LCL was not possible.

To provide a useful indirect model-data comparison, however, we define a region within lowland Amazonia, removing highland grid cells so that elevation does not become a confounding variable in our LCL analysis. Consistent with Hahn & Warren (2002), we excluded all highland regions located along the eastern flank of the Andes Mountains (i.e. grid cells where mean annual temperature less than 21°C) and all oceanic grid points. Because the observational data exhibited very little seasonal variation (statistically not significant), we used the four 3-month averages to generate annual averages for comparison against our calculated annual LCL values.

## 3. Results and discussion

### (a) Data-model comparisons

The majority of our lowland area has model-calculated LCL values that vary between 650 and 850 m (Pinto *et al*. in press). While the observed LCL is 771±28 m, our model-predicted LCL for the lowland region is 832±20 m, a difference of approximately 61 m. Two-tailed *z*-statistics show that the means between datasets do not significantly differ (*p*=0.3734, *α*=0.05). When we consider both the uncertainty associated with modelling cloud formation at GCM scales (Still *et al*. 1999; Henderson-Sellers 2006) as well as the typical margin of error of more than 500 m in estimating cloud base height in meteorological observations (Craven *et al*. 2002), our simulated LCL closely approximates observations.

Model-predicted value of vegetation WRE near the start of our simulation (i.e. AD 2000) is 0.65 in our coupled scenario. Comparison against calculated recycling efficiencies from eddy-flux data from predominantly old-growth tropical rainforest in the neotropics (table 1) indicates similar values, however our GCM-based values are nearer to the higher end of the calculated range (range: 0.64–0.42, mean=0.52±0.07).

### (b) Water-recycling efficiency in Amazonia

Decreased broadleaf cover from 85 to 50% leads to a 33% decrease in the rate of P in our coupled relative to uncoupled simulation (figure 1*b*). At the end of the modelling experiment (i.e. simulation year AD 2090), the overall difference in P between simulations is approximately 1 mm d^−1^. ET is over 50% lower by the end of the modelling experiment in the coupled scenario, yet only 30% reduced in the uncoupled simulation (table 2).

Considerable literature focuses on the degree to which highly productive rainforests can return water to the atmosphere via transpiration, including the evaporation of water pooling on leaf canopy surfaces (Kleidon & Heimann 2000; Notaro *et al*. 2007). Estimates have ranged from as low as 25% (Brubaker *et al*. 1993) to more than 50% (Leopoldo *et al.* 1987). Discrepancies between the estimates primarily stem from difficulties in estimating ET in the field, which in turn are limited by our inability to fully understand the complex network of underlying climatic and physiological mechanisms influencing ET.

Calculated WRE (ET/P) of Amazonian vegetation reveals a mean value of 0.65±0.01 for our coupled simulation and 0.69±0.01 for uncoupled simulation. The coupled simulation shows a lower WRE because the inclusion of stomatal physiology results in a proportionally greater ET decline relative to P as a result of regional warming. Consequently, WRE does not show an over trend through time (figure 2). WRE in the year 2000 is already lower in the coupled relative to uncoupled simulation (despite similar percent broadleaf cover) because increases in atmospheric CO_2_ begin at the start of the Industrial Revolution (*ca* 1870 AD), resulting in a trend towards stomatal closure (i.e. improved plant water-use efficiency).

Dekker *et al*. (2007) employ models of varying scales to show that micro-scale vegetation feedbacks on climate (i.e. increased P promoting increased vegetation, leading to increased soil infiltration that in turn causes greater vegetative growth) are as important as macro-scale vegetation feedbacks on climate like ET–P coupling. In addition, our results indicate that the WRE of tropical rainforests such as the Amazon Basin may remain relatively constant despite a change in climate. This trend probably arises from the tight relationship between vegetative surface area (i.e. per cent broadleaf cover) and rate of ET (figure 3*a*; *r*^2^=0.95) together with the close relationship between ET and P (figure 3*b*; *r*^2^=0.997 for coupled and *r*^2^=0.984 for uncoupled simulations). As ET decreases with declining broadleaf cover in response to warming climate, so does P, yielding a relatively constant ratio of ET to P over time.

### (c) Vegetation feedbacks on surface energy balance: implications for cumulus cloud formation

Between the start and the end of our simulation, sensible heat (*Q*_H_) increases by more than 100% and by less than 60% in the coupled and uncoupled simulations, respectively (table 2). Bowen ratio increases monotonically with increasing time (i.e. future climate warming and drying) for both our coupled and uncoupled simulations, although the rate of change is much steeper in the coupled scenario (figure 4*b*). Similarly, the slope of the plot of coupled per cent broadleaf cover versus Bowen ratio (*Q*_H_/*Q*_E_) shows that the rate of change of Bowen ratio calculated when per cent broadleaf cover is high is twice as much as that calculated for lower values of per cent broadleaf cover (slope=0.040 and 0.017 respectively; figure 4*a*). The threshold defining these responses lies somewhere around 70% broadleaf cover.

The effect of decreasing vegetation cover on surface energy and water exchange has been the focus of several modelling experiments (Freedman *et al*. 2001; Ray *et al*. 2003; Crucifix *et al*. 2005; Simon *et al*. 2005; Sogalla *et al*. 2006; Betts *et al*. 2007; Dekker *et al*. 2007). A recent study by Crucifix *et al*. (2005) show that dynamic vegetation in a fully coupled GCM substantially increases the variability of surface *Q*_H_ and *Q*_E_ fluxes at the *global* scale, and that increased vegetation cover always increases ET; the latter conclusion supported by our simulations (figure 3*a*). We postulate two trends in response of surface energy–water balance to variations in forest cover: (i) above a vegetation fraction threshold (in our case, approx. 70% broadleaf cover), physiological factors like the effects of stomatal conductance on rate of ET play an important role in modifying Bowen ratio and (ii) below this threshold (i.e. progressively less forest cover), other factors like surface albedo begin to dominate the partitioning of surface energy.

As expected, both coupled and uncoupled simulations show tight correlations between LCL and ET (figure 5*a*). However, at the end of our simulation experiment (*ca* AD 2100), LCL is over 520 m higher in the coupled relative to uncoupled simulation (figure 5*b*), clearly demonstrating vegetation feedbacks on cumulus cloud formation. Any change in LCL between simulations must be due to the fact that vegetation is either fixed or dynamic as no other variable is different between simulations. In support, the statistical correlation between LCL and per cent broadleaf cover yields a correlation coefficient that is relatively high (*r*^2^=0.94; figure 5*c*). Modifications to LCL in our coupled simulation probably results from two biophysical–physiological processes: (i) the amount of moisture that lowland Amazonia recycles back to the atmosphere via ET (figure 3*a*) and (ii) the amount of warming that is promoted when decreasing vegetation cover partitions more incoming solar energy into *Q*_H_ relative to *Q*_E_ energy (i.e. in increasing Bowen ratio with decreasing per cent broadleaf cover; figure 4*a*). Both of these plant-based processes (decreased rate of ET, increasing Bowen ratio) promote an upward displacement of LCL.

Although this study does not directly address factors related to canopy height and architecture, called *roughness length* (i.e. the degree of landscape heterogeneity), they can also strongly modify regional cloud formation (Salmun & Molod 2006). Our modelling simulations include feedbacks from surface roughness on the depth of convective boundary layer (i.e. described in Hadley's land-surface model, MOSES). This means that our simulated (calculated) LCL results from changes in roughness element, in addition to surface temperature, surface pressure and specific humidity (table 2).

The role of vegetation feedbacks on LCL has been shown by others (O'Neal 1996; Emori 1998; Still *et al*. 1999; Ray *et al*. 2003; Ek & Holtslag 2004). Using a regional land-surface model, Emori (1998) and Ek & Holtslag (2004) show that changes in surface temperature arising from variations in evaporation cause large contrasts in soil moisture that in turn strongly influence thermally induced cumulus convection. MODIS data between 1999 and 2000 for southwest Australia reveal a higher frequency of cumulus clouds and a greater optical density of clouds over native vegetation relative to agricultural fields during the dry summer (Ray *et al*. 2003). Satellite data between latitudes 35 and 55° N over eastern North America show greater convective cloud cover over forests in Ontario and the Appalachians relative to areas over the Great Lakes (O'Neal 1996).

## 4. Summary

We present a very basic, yet statistically direct, analysis of the effects of Amazonian vegetation in influencing WRE that in turn modify both LCL and regional rates of precipitation. The simplicity in our modelling experiments stems from the fact that the only difference between simulations is the presence or the absence of dynamic vegetation feedbacks onto climate. We show that WRE in Amazonia remains relatively conserved over changing climate primarily owing to the tight correlation between ET and per cent broadleaf cover and between ET and P. The rate of change of Bowen ratio with per cent broadleaf cover is the greatest at high fractional coverage (i.e. above 70% broadleaf cover). Over the duration of our modelling experiment, P is reduced and LCL increased by 52 and 55% (respectively) in our coupled simulation, whereas in our uncoupled simulation P decreases and LCL increases by only 28 and 37% (respectively).

## Figures and Tables

**Figure 1:**
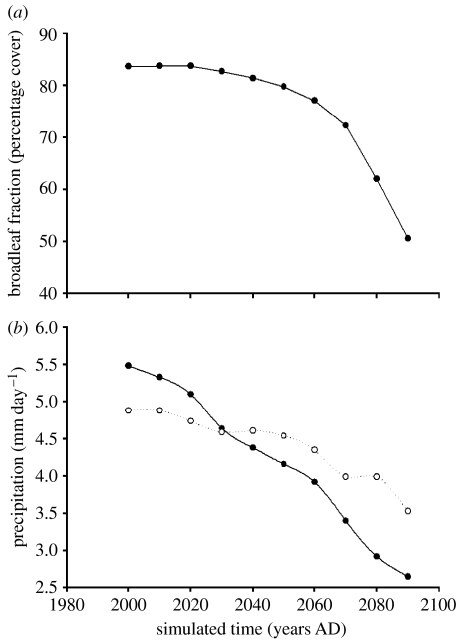
Response of (*a*) broadleaf cover (percentage of surface area) to changes in climate defined by IPCC's IS92a CO_2_ emission scenario (coupled simulation) and (*b*) precipitation (mm d^−1^) versus simulation time (years AD) for our coupled and uncoupled simulations. Per cent broadleaf cover remains fixed (approx. 84%) throughout our uncoupled simulation. Filled circles, time versus P-coupled stimulation; open circles, time versus P-uncoupled stimulation.

**Figure 2:**
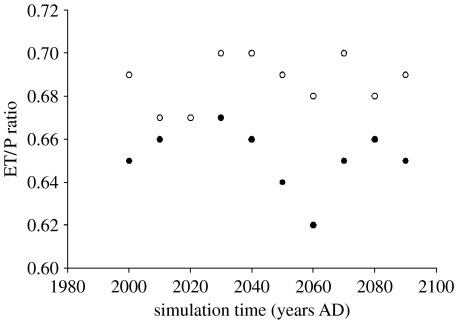
Plotted relationship for the ratio of evapotranspiration (ET, mm d^−1^) to total precipitation (P, mm d^−1^) versus simulation time (years AD).

**Figure 3:**
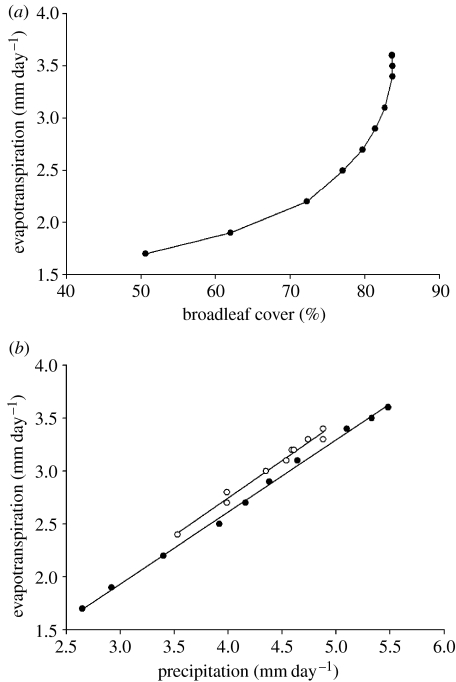
Plotted graph of (*a*) rate of ET (mm d^−1^) versus per cent broadleaf cover for our coupled simulation (*r*^2^=0.95) and (*b*) rate of ET (mm d^−1^) versus precipitation (mm d^−1^) for both coupled (*r*^2^=0.997; filled circles, P versus ET-coupled) and uncoupled (*r*^2^=0.984; open circles, P versus ET-uncoupled) simulations. Solid lines, regression plot.

**Figure 4:**
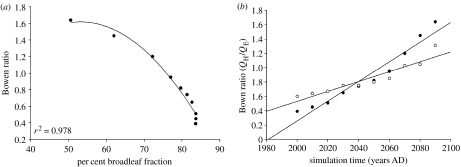
Graphed relationship between (*a*) Bowen ratio and per cent broadleaf cover for our coupled simulation (*r*^2^=0.978) and (*b*) Bowen ratio and simulation time (years AD) for both coupled (*r*^2^=0.946; filled circles) and uncoupled (*r*^2^=0.886; open circles) simulations. Filled circles, time versus B-coupled simulations; open circles, time versus B-uncoupled simulations; solid lines, regression plot.

**Figure 5:**
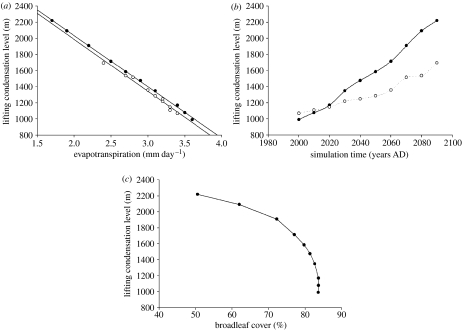
Plotted relationship for (*a*) LCL (m) versus ET (mm d^−1^) for both coupled (filled circles, ET-coupled versus LCL-coupled) and uncoupled (open circles, ET-uncoupled versus LCL-uncoupled) simulations; solid lines, plot 1 regression, (*b*) LCL (m) versus simulation time (years AD) for both coupled (filled circles, time versus LCL-coupled) and uncoupled (open circles, time versus LCL-uncoupled) simulations; and (*c*) LCL (m) versus per cent broadleaf cover for our coupled simulation.

**Table 1:** Summary of field-based observations of mean annual evapotranspiration (ET, mm yr^−1^) and precipitation (P, mm yr^−1^) for Neotropical forests including both undisturbed rainforest and plantations. Calculated ET/P for listed studies equals 0.52±0.11.

P (mm yr^−1^)	ET (mm yr^−1^)	ET/P	site location	reference
2200	1300	0.59	Tapajos, Brazil	da Rocha *et al*. (2004)
2089	1124	0.53	Caieiras, Brazil	Malhi *et al*. (2002)
2892	1481	0.51	Janlappa, Java	Calder *et al*. (1986)
4620	2172	0.47	La Selva, Costa Rica (old growth)	Loescher *et al.* (2005)
3156	1318–1509	0.42–0.48	La Selva, Costa Rica (plantation)	Bigelow (2001)
3495	1892	0.54	Costa Rica (1998)	Loescher *et al*. (2005)
3575	2294	0.64	Costa Rica (1999)	Loescher *et al*. (2005)
4127	2239	0.54	Costa Rica (2000)	Loescher *et al*. (2005)

**Table 2:** Latent heat flux (W m^−2^), sensible heat flux (W m^−2^), surface temperature (°C), specific humidity (g kg^−1^) and surface pressure (mbar) for both coupled (CPL) and uncoupled (UNC) simulations.

time (AD)	latent heat flux (W m^−2^)		sensible heat flux (W m^−2^)		temperature (°C)		sp. humidity (g kg^−1^)		surface pressure (mbar)	
				
	CPL	UNC	CPL	UNC	CPL	UNC	CPL	UNC	CPL	UNC
2000	104	98	40.8	59.0	30	31	17.3	17.4	1115.2	1115.8
2010	100	96	45.2	61.1	31	31	17.4	17.4	1114.8	1115.7
2020	97	95	49.3	63.8	32	32	17.4	17.5	1114.8	1115.7
2030	89	92	57.6	67.5	33	32	17.31	7.7	1114.5	1115.5
2040	84	91	62.3	68.2	34	33	17.4	17.8	1114.3	1115.4
2050	79	89	65.1	71.0	35	33	17.3	18.0	1114.3	1115.4
2060	73	87	69.5	73.7	36	34	17.3	17.9	1114.1	1115.3
2070	64	80	77.4	82.3	38	35	17.4	18.2	1113.6	1114.8
2080	56	79	80.9	83.1	39	35	17.2	18.0	1113.6	1114.8
2090	50	71	82.0	92.7	40	37	17.4	18.1	1113.4	1114.5
